# An alternative covariance estimator to investigate genetic heterogeneity in populations

**DOI:** 10.1186/s12711-015-0171-z

**Published:** 2015-11-26

**Authors:** Nicolas Heslot, Jean-Luc Jannink

**Affiliations:** Department of Plant Breeding and Genetics, Cornell University, 240 Emerson Hall, Ithaca, NY 14853 USA; Limagrain Europe, CS3911, 63720 Chappes, France; USDA-ARS, R.W. Holley Center for Agriculture and Health, Cornell University, Ithaca, NY 14853 USA

## Abstract

**Background:**

For genomic prediction and genome-wide association studies (GWAS) using mixed models, covariance between individuals is estimated using molecular markers. Based on the properties of mixed models, using available molecular data for prediction is optimal if this covariance is known. Under this assumption, adding individuals to the analysis should never be detrimental. However, some empirical studies showed that increasing training population size decreased prediction accuracy. Recently, results from theoretical models indicated that even if marker density is high and the genetic architecture of traits is controlled by many loci with small additive effects, the covariance between individuals, which depends on relationships at causal loci, is not always well estimated by the whole-genome kinship.

**Results:**

We propose an alternative covariance estimator named K-kernel, to account for potential genetic heterogeneity between populations that is characterized by a lack of genetic correlation, and to limit the information flow between a priori unknown populations in a trait-specific manner. This is similar to a multi-trait model and parameters are estimated by REML and, in extreme cases, it can allow for an independent genetic architecture between populations. As such, K-kernel is useful to study the problem of the design of training populations. K-kernel was compared to other covariance estimators or kernels to examine its fit to the data, cross-validated accuracy and suitability for GWAS on several datasets. It provides a significantly better fit to the data than the genomic best linear unbiased prediction model and, in some cases it performs better than other kernels such as the Gaussian kernel, as shown by an empirical null distribution. In GWAS simulations, alternative kernels control type I errors as well as or better than the classical whole-genome kinship and increase statistical power. No or small gains were observed in cross-validated prediction accuracy.

**Conclusions:**

This alternative covariance estimator can be used to gain insight into trait-specific genetic heterogeneity by identifying relevant sub-populations that lack genetic correlation between them. Genetic correlation can be 0 between identified sub-populations by performing automatic selection of relevant sets of individuals to be included in the training population. It may also increase statistical power in GWAS.

**Electronic supplementary material:**

The online version of this article (doi:10.1186/s12711-015-0171-z) contains supplementary material, which is available to authorized users.

## Background

Genomic prediction (GP) is now widely used in plants and animals. Numerous methods have been developed but a common approach is the GBLUP (genomic best linear unbiased prediction) model that uses markers to estimate the covariance between the performances of individuals. Similar models are also used for genome-wide association studies (GWAS) to control population structure and background effects in the detection of significant marker-trait associations e.g., [1]. Many genomic prediction studies showed that the prediction accuracy of the GBLUP model decreases as more individuals are added to the training population. In animal breeding, this problem has received considerable attention in the context of prediction between breeds (reviewed in [2]) and, so far, empirical results obtained with the GBLUP model have been disappointing. Hayes et al. [3] showed that the expected accuracies that were derived from the mixed model matched the within-breed observed accuracies but not the between-breed observed accuracies, and poor predictive ability was observed from one breed to the other. Similar poor between-breed accuracies were reported by Daetwyler et al. [4] who also showed that most of the observed accuracy was due to population structure. In goats, Carillier et al. [5] reported small gains in accuracy with multi-breed GBLUP compared to pedigree-based BLUP, which does not use information between breeds.

In plants, similar results have been reported. Windhausen et al. [6] found no within full-sib family prediction ability in maize when the training population was a panel of unrelated individuals. Adding more distantly related individuals was either not helpful or detrimental. In a set of maize connected crosses, composed of large full-sibs families, Riedelsheimer et al. [7] showed that prediction followed expectations when training and validation individuals were in the same family but not otherwise. In wheat, Dawson et al. [8] used historical data from international nurseries that were collected between 1992 and 2009, and reported inconsistent accuracies when they used data from previous years to predict accuracies of later years. These prediction accuracies were not explained by variation in the quality of the phenotype data of the training or validation sets. Finally, Rutkoski et al. [9] showed that accuracies were lower with a training population of 365 individuals than with optimized subsets of that population that were less than half its size.

If the covariance between individuals was well estimated by the current models, adding more individuals to the analysis should never be detrimental [10]. Consequently, the empirical results are evidence that the true covariance matrix between individuals, **G**, is not always well estimated by the whole-genome kinship. The GBLUP estimate of **G** using $$m$$ biallelic markers, $${\hat{\mathbf{G}}}$$, is based on $${\mathbf{W}}$$, the $$n\; \times \;m$$ centered marker score matrix coded as $$\left. {\left\{ {BB,\;Bb,\;bb} \right.} \right\} = \left. {\left\{ {2 - 2p,\;1 - 2p,\; - 2p} \right.} \right\}$$ with $$p$$ the frequency of allele $$B$$ and $${\hat{\mathbf{G}}} = \gamma {\mathbf{S}}$$ with $${\mathbf{S}} = {\raise0.7ex\hbox{${{\mathbf{WW}}^{\prime}}$} \!\mathord{\left/ {\vphantom {{WW^{\prime}} {2\sum\limits_{j} {p_{j} \left( {1 - p_{j} } \right)} }}}\right.\kern-\nulldelimiterspace} \!\lower0.7ex\hbox{${2\sum\limits_{j} {p_{j} \left( {1 - p_{j} } \right)} }$}}$$ [11]. $$\gamma$$ is a variance component estimated by restricted maximum likelihood (REML) [4].

If there are too few markers, this estimator is suboptimal and some shrinkage is needed to better estimate the whole-genome relationship [12–14]. Two non-mutually exclusive mechanisms may lead to poor covariance estimation in the presence of high-density markers or even full-sequence data. First, at high marker density, ${\hat{\mathbf{G}}} = \gamma {\mathbf{S}}$ will perfectly describe the whole-genome relationship, but **G** depends only on the causal loci [15]. As a consequence, even if all the polymorphisms present in the population are known through whole-genome sequencing, the whole-genome relationship can be perfectly calculated but **G** will still be only estimated. As shown by Hill and Weir [16], **G** is not well estimated by ${\hat{\mathbf{G}}}$ for distantly-related individuals, even if marker density is high and for additive traits controlled by many loci. Only for a truly infinitesimal trait (all loci affect the trait) and when ${\hat{\mathbf{G}}}$ is calculated on the basis of the whole-genome sequence should ${\mathbf{G}}$ and ${\hat{\mathbf{G}}}$ converge. Needless to say, this situation exists only in thought experiments, but it suggests that this effect will depend on effective population size and trait architecture. A second mechanism is genetic heterogeneity between distantly-related individuals. Conceptually, such individuals belong to different populations, and, for quantitative traits, it is assumed that genetic heterogeneity between populations occurs when causal loci or alleles that affect a trait differ, or their gametic phase disequilibrium with nearby polymorphisms differs, between populations. In practice, these two mechanisms are not easy to distinguish since their impacts are consistent with each other.

This discussion makes clear that improved estimation of **G** is a critical issue for the design of training populations, for increased prediction accuracy and for increased power of the detection of marker-trait associations. De los Campos et al. [17], showed that finding an estimator for ${\mathbf{G}}$ or kernel has received much attention in the machine learning literature under the name “reproducing kernel Hilbert spaces” (RKHS), which was recently reviewed in [18]. A large body of theory indicates that ${\hat{\mathbf{G}}} = \gamma {\mathbf{S}}$ is one of many possible kernels and shows that the genetic analysis issue can be reduced to the choice of a kernel function to estimate the covariance between individuals based on markers. Each kernel function defines a family of kernels indexed by a number of parameters. It can be shown that the kernel function or family selected determines the functional form of all possible solutions considered [19].

Our first objective was to propose a multiple kernel model to study the problem of genetic heterogeneity without pedigree or a priori knowledge about subpopulations. The second objective was to compare the new model to other available kernels using empirical data.

## Theory

### Mixed models

A basic mixed model for genetic evaluation or GWAS can be written as:

$${\mathbf{y = X\boldsymbol{\beta} + Zu + \boldsymbol{\epsilon} }},$$

where ${\mathbf{y}}$ is a vector of phenotypes, ${\varvec{\upbeta}}$ is a vector of fixed effects, ${\mathbf{u}}$ is a vector of random individual effects normally distributed with mean $0$ and covariance ${\mathbf{G}}$ and ${\varvec{\boldsymbol{\epsilon}}}$ is a vector of i.i.d. errors with common variance $\sigma_{e}^{2}$, which is a common approximation. ${\mathbf{X}}$ and ${\mathbf{Z}}$ are design matrices for the fixed and random effects, respectively. For GWAS, the fixed effects ${\mathbf{X}}$ will contain the marker for which the effect is tested and possibly some covariates to further control population structure [1]. Those covariates are often eigenvectors of a principal component analysis of the marker data and form a so-called ${\mathbf{Q}}$ matrix. This model produces best linear unbiased prediction (BLUP) for the random effect ${\mathbf{u}}$ assuming that the variance components are known [10] which is not usually the case. To estimate ${\mathbf{G}}$, there are alternative kernels to replace ${\mathbf{S}}$, as described below.

### Common kernels in genetic evaluation

De los Campos et al. [17] showed that the genetic evaluation problem could be viewed as a kernel estimation and selection problem. If markers are not available, ${\mathbf{S}}$ can be replaced by the numerator relationship matrix based on pedigree records ${\mathbf{A}}$.

Another common family of kernels is the Gaussian family. In that case, ${\mathbf{S}}$ is replaced by **U** such that ${\mathbf{U}}_{ij} = \exp ( - ({{{\mathbf{D}}_{ij} } \mathord{\left/ {\vphantom {{{\mathbf{D}}_{ij} } \theta }} \right. \kern-0pt} \theta })^{2} )$, where the matrix of Euclidean distances between individuals ${\mathbf{D}}$ is calculated with markers and normalized to the interval [0, 1] and $\theta$ is a so-called bandwidth parameter estimated by REML using a grid search. The Gaussian kernel is widely used in machine learning [19]. For fully inbred individuals, when $\theta$ is large relative to ${\mathbf{D}}$, ${\mathbf{U}}$ is equal to ${\mathbf{S}}$ [20]. A Taylor series expansion of the Gaussian kernel can be written: ${\mathbf{U}}_{ij} = 1 - ({{{\mathbf{D}}_{ij} } \mathord{\left/ {\vphantom {{{\mathbf{D}}_{ij} } \theta }} \right. \kern-0pt} \theta })^{2} + {1 \mathord{\left/ {\vphantom {1 2}} \right. \kern-0pt} 2}({{{\mathbf{D}}_{ij} } \mathord{\left/ {\vphantom {{{\mathbf{D}}_{ij} } \theta }} \right. \kern-0pt} \theta })^{4} + \cdots$ when $\theta$ is large relative to ${\mathbf{D}}$ [20]. This shows that as $\theta$ decreases, higher order interactions become more important, and suggests that the Gaussian kernel can capture some epistasis non-parametrically for fully inbred individuals. As a consequence, the random effect predictions are genotypic and not breeding values. The Gaussian kernel tends to be slightly more predictive of phenotype than ${\mathbf{S}}$ on empirical data [18]. For outbred populations, there is no demonstration beyond the fact that RKHS is a universal approximator that will seek to fit the genotypic value fully, including additive and non-additive components. As such, it should be able to capture some proportion of the epistatic variance, if there is any.

When the number of markers is small compared to the number of individuals, Endelman et al. [14] have shown that ${\mathbf{S}}$ is not a good estimator of the whole-genome kinship and can be replaced by a shrunken estimate such that ${\mathbf{V}} = \delta {\mathbf{T}} + (1 - \delta ){\mathbf{S}}$ with ${\mathbf{T}}$, a so-called low-dimensional target. In their paper, they used ${\mathbf{T}} = \left\langle {S_{ii} } \right\rangle {\mathbf{I}}$ with $\left\langle {S_{ii} } \right\rangle$ being the average of the diagonal elements of ${\mathbf{S}}$, which makes the shrunken estimate an unbiased estimator in the sense that ${\mathbf{V}}$ is equal to ${\mathbf{S}}$ at high marker density. ${\mathbf{V}}$ minimizes the mean squared error with the covariance between individuals computed using an infinite number of markers. $\delta$ is a shrinkage parameter that is calculated using an estimator from [12]. If $\delta = 0$, there is no shrinkage and ${\mathbf{V}}$ corresponds to ${\mathbf{S}}$. If $\delta$ is greater than 0, the off-diagonal elements of ${\mathbf{S}}$ are shrunk towards $0$. The shrinkage intensity does not depend on phenotype but on the number of markers relative to the number of individuals.

Similarly, Goddard et al. [13] argued that ${\mathbf{S}}$ is biased compared to ${\mathbf{A}}$, the numerator relationship matrix, because of a finite number of markers and proposed to use ${\mathbf{A}}$ itself as a target ${\mathbf{T}}$. They also proposed an estimator of $\delta$ that produces no shrinkage if the number of markers is very large relative to the number of individuals. Both kernels are not used in this paper.

In the GWAS context, another kernel function was proposed by [21] and termed compressed mixed model. The idea is to cluster individuals based on ${\mathbf{S}}$ and use the clusters themselves as random effects instead of the individuals. The covariance between clusters is obtained by averaging the elements of ${\mathbf{S}}$ within and between clusters. This reduces computing time by reducing the number of levels of random effects and, in their simulations, it increased statistical power compared to the use of a GBLUP model [21]. However, it seems that some information is lost by averaging elements of ${\mathbf{S}}$ within clusters. In the following, we name this kernel family “C-kernels”. Note that the C-kernel is identity within populations (clusters), so it has no ability to predict or correct (for GWAS) within populations.

### K-kernels

All the kernels presented above have a common limitation: they do not allow for genetic heterogeneity. For example, they cannot describe well a situation where two populations have completely different additive genetic architectures. Such a case would be best described by a block diagonal covariance structure. Recently, [22, 23] proposed a multi-trait solution, if groups are known a priori. Here, we propose a similar model without prior identification of the groups and without using pedigree information, which we named “K-kernels” indexed by two parameters $k$ and $h$ such that ${\mathbf{K}} = h{\mathbf{S}}_{{\mathbf{k}}} + (1 - h){\mathbf{S}}$. $h$ is a mixture parameter that varies between $0$ (GBLUP model) and 1 (block-diagonal covariance reduced to the target ${\mathbf{S}}_{{\mathbf{k}}}$). ${\mathbf{S}}_{{\mathbf{k}}}$ is a block diagonal matrix with individuals ordered into $k$ clusters, if individuals $i$ and $j$ belong to the same cluster, the corresponding element of ${\mathbf{S}}_{{\mathbf{k}}}$ is equal to the element of ${\mathbf{S}}$ and 0 otherwise. For a given $k$, the population is split in $k$ clusters that are determined by transforming ${\mathbf{S}}$ in a distance matrix and using a classical clustering algorithm. That is for a given $k$, the assignment of individuals to each cluster $k$ is done by a clustering algorithm. The values of $k$ and $h$ are estimated by REML using a grid search. It can be seen as a multiple-kernel model or a simplified multi-trait model. A similar idea was also proposed by [11].

This means that the proposed K-kernels allow in one extreme case, $k$ populations, each with an independent additive trait architecture, and, in the other extreme case, a single population with an additive model that is equivalent to the GBLUP model. Intermediate values of $h$ enable some genetic correlation between populations. This model attempts to better estimate the true covariance between individuals.

## Methods

### Data

We used several publicly available datasets representative of plant and animal breeding data, which are summarized in Table 1. Additional information is available in Additional file 1: Table S1.

**Table 1:** Summary of the datasets used

Name	Species	Description	Traits
Loblolly pine	Loblolly pine [33]	926 individuals, 5000 SNPs	Five wood quality traits
Cimmyt wheat	Wheat [34]	599 individuals, 1279 DArT markers	Yield in four environments
Pig	Pig [35]	3460 individuals, 53k SNPs	Two anonymous trait
Maize panel	Maize [28]	2279 inbreds, 68,120 GBS SNPs imputed	Flowering time in degree days
Maize connected crosses	Maize [7]	635 inbreds, 17k SNPs	Five traits (two diseases, three yield components)
Cornell wheat	Wheat [36]	Breeding population 365 individuals, 32k GBS SNPs imputed	Four traits (yield, height, heading date, pre-harvest sprouting)
Rice panel	Rice [27]	398 individuals, diverse panel 36,901 SNPs	28 traits (flowering time, yield components and quality traits)

*SNP* single nucleotide polymorphisms, *GBS* genotyping by sequencing, *DArT* diversity arrays technology

### Kernel comparison

For each dataset and trait, GBLUP, Gaussian kernel, C-kernel and K-kernel models were fitted to all the data available using the R package rrBLUP [24]. For the C- and K-kernels, after transformation of ${\mathbf{S}}$ into a distance matrix, individuals were clustered using either Ward’s minimum variance criterion, which yields compact clusters or UPGMA (unweighted pair group method with arithmetic mean) as suggested by [21]. The kernel parameters $\theta$ for the Gaussian kernel as well as $k$ and $h$ for the K-kernel and $k$ for the C-kernel were estimated by REML using a simple grid search with *k* ranging from 1 to 15 for K-kernel, from 5 up to the number of individuals by steps of 5 for the C-kernel, and $h$ from 0 to 1 by steps of 0.1. For a given $k$, affiliation of each individual to a cluster was done using either one of the clustering methods described above.

The GBLUP kernel is nested within the Gaussian, K- and C-kernels. For inbred individuals, if $\theta = 1$, the Gaussian kernel reduces to GBLUP (here $D$ is normalized to [0, 1]). With the C-kernel, if the number of clusters $k$ is equal to the number of individuals, it reduces to GBLUP. Finally, for the K-kernel, if $h = 0$ for any k, it reduces to GBLUP. Alternative kernels were compared to GBLUP using a likelihood ratio test with one degree of freedom for the Gaussian and C-kernels. For the K-kernel, two degrees of freedom were counted to account for two additional parameters compared to the GBLUP. Because the hypothesized parameters are placed on the boundary of the parameter space, those tests will be overly conservative [25]. The impact of the clustering method on the test is also unclear. The validity of the likelihood ratio test was tested for each trait and dataset by generating 100 samples of breeding values from a multivariate normal distribution with covariance $\gamma {\mathbf{S}}$, fitting the different kernels and calculating the likelihood ratio test statistics. This generated an empirical null distribution to confirm the validity of the likelihood ratio test. This likelihood-ratio test examines whether any of the alternative kernels describe the true genetic architecture of the trait significantly better. In particular for the K-kernel, the significance of genetic heterogeneity and its extent are tested.

To select the most predictive model, the AIC (Akaike information criterion) was used to compare kernels along with cross-validated prediction accuracy. The AIC quantifies the goodness of prediction rather than goodness of fit [26]. The number of effective parameters used was the number of variance components and kernel parameters, as for the likelihood ratio test. Alternative formulations of the AIC were also tested but did not improve the results (results not shown). Cross-validation was 10-fold, iteratively masking 10 % of the individuals and predicting them with the different models. For each fold, the different models were fitted to the data, parameters were identified as for the complete datasets and the performance of masked individuals was predicted. Cross-validation sampling was done in two ways. In the first case, sampling was stratified across the different clusters to ensure that any validation set was representative of the population. In the other cross-validation, the clusters with $k = 10$ obtained with Ward’s criterion were used as cross-validation partitions, testing the ability of the models to discriminate between closely-related individuals. If the AIC predicts observed cross-validated accuracy, it will indicate that the model does not overfit the data and can be used with confidence for genomic prediction. On the contrary, if the AIC does not predict observed cross-validated accuracy, it suggests that the model overfits the data. Correlations between AIC and cross-validated accuracies were calculated.

### GWAS

For the rice [27] and maize panels [28], which are both diversity panels used for extensive GWAS, we compared the classical GBLUP to the Gaussian, C- and K-kernels to correct for population structure. The P3D approach of [21] was used, in which variance components are determined just once to speed up computation. This included estimation of $\theta$, $k$ and $h$ by grid search using REML once for each trait. Quantile–Quantile (QQ) plots were used to compare type I error (false positive) rates between the models [1]. In all cases, the models included a ${\mathbf{Q}}$ matrix of fixed effects that accounted for population structure, which was chosen to be identical to that used in the initial GWAS for both datasets. For the C-kernel model, this might seem like double-counting but the structure of ${\mathbf{Q}}$ was taken as input and fitted as fixed effect for each model, to be able to compare the log-likelihood between the different models.

### GWAS simulations

To assess the statistical power of GWAS with the different kernels, we used the simulation scheme of [21] which preserves the original architecture of the trait. Five thousand markers were sampled as potential causal quantitative trait nucleotides (QTN). For each trait analyzed with the rice and maize datasets, and iteratively for each sampled marker, a true QTN effect was added to the observed phenotype. GWAS was carried out to test if the true QTN could be detected, using the P3D approach with $\gamma$, $\theta$, $k$ and $h$ previously determined on the real data. The added true QTN effect contributed only a small proportion of the original phenotypic variance and was determined as a fraction $q$ of the original phenotypic standard deviation between 0.1 and 0.5. This true QTN would explain at most 11.1 % of the phenotypic variation depending on the frequency $f$ of the true QTN in the population. The percentage of total variation explained by the true QTN is $1/(1 + 1/2f(1 - f)q^{2} )$ with $f$ being the frequency of the true QTN as described in [21]. Statistical power was calculated as the percentage of true QTN detected at the significance threshold 0.01.

All computations were implemented using R 2.15.3 [29].

## Results

### Comparison of kernels

The empirical null distributions of the log-likelihood ratio test for each trait and dataset allowed the verification of the test assumptions and the control of type I errors based on the observed 95 % quartile. For the Gaussian kernel, the mean of the 95 % quartile of the simulated likelihood ratio test statistics was equal to 2.58 with a minimum of 0 and a maximum of 3.84 across traits and datasets, which was close to the expected value of 3.84 for the 95 % quartile of the Chi squared distribution with one degree of freedom (one additional parameter). Thus, test assumptions were correct for the Gaussian kernel. For the K-kernel, the mean was equal to 4.70 with a minimum of 3.55 and a maximum of 5.74 when the 95 % quartile of the Chi squared distribution with two degrees of freedom (two additional parameters) was equal to 5.99. Thus, test assumptions were correct for the K-kernel. For the C-kernel although it requires only one additional parameter, the mean was equal to 4.71 with a minimum of 3.2 and a maximum of 5.94 when a value of 3.84 for the 95 % quartile is expected. Thus, test assumptions were incorrect for the C-kernel. In the following, for the C-kernel, the likelihood ratio test was corrected to account for two parameters to better control for false positives. The AIC of the C-kernel was also corrected to account for two parameters instead of one.

**Fig. 1:**
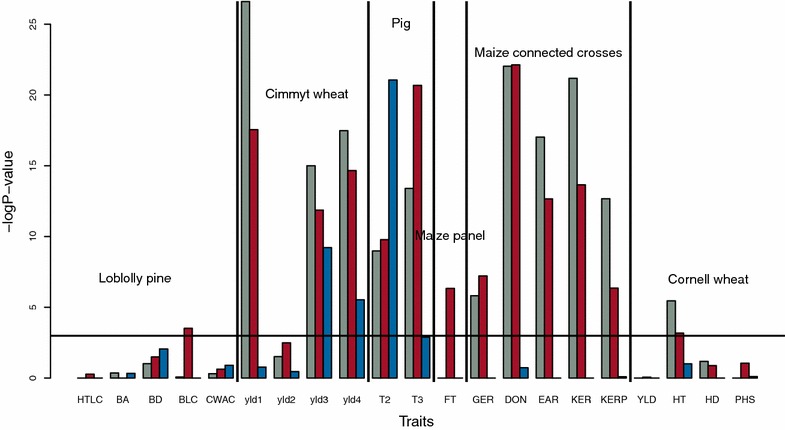
Minus log P values of the log-likelihood ratio tests for the Gaussian kernel (*gray*), K-kernel (*red*) and C-kernel (*blue*) for each trait and dataset except the rice dataset (see Fig. 2). The *horizontal line* indicates the significance level at 0.05 with Bonferroni correction for multiple testing on trait-dataset combinations; the *vertical lines* separate the different datasets; datasets are presented in Table 1 and more details on the traits are available in Additional file 1: Table S1

**Fig. 2:**
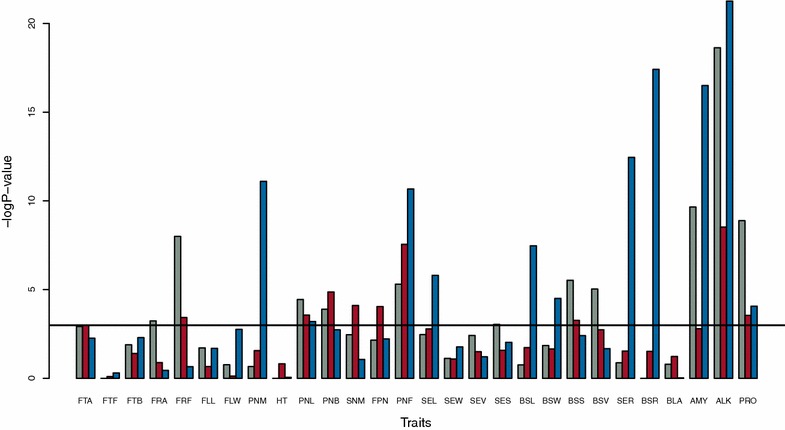
Minus log P values of the log-likelihood ratio tests for the Gaussian kernel (*gray*), K-kernel (*red*) and C-kernel (*blue*) for each trait of the rice dataset. The *horizontal line* indicates the significance level at 0.05 with Bonferroni correction for multiple testing; the rice dataset is in Table 1 and more details on the traits are available in Additional file 1: Table S1

Figures 1 and 2 present the results of the log-likelihood ratio test for each trait and dataset for the Gaussian, K- and C-kernels. The log-likelihood ratio test tests if the alternative kernels describe the genetic architecture of the traits considered significantly better than GBLUP. The horizontal bar indicates the 5 % significance threshold if a Bonferroni correction for multiple-testing is applied on trait-dataset combinations. A lower *P* value, corresponding to a high negative log of P-value indicates that an alternative kernel describes the genetic architecture significantly better than GBLUP. This is the case for a number of traits, even after Bonferroni correction. The C-kernel performed well on the CIMMYT wheat dataset that is characterized by a small number of markers and on the rice dataset (Fig. 2). The Gaussian kernel had a very significantly improved fit to the data for the pig population, maize connected crosses and CIMMYT wheat datasets. Similarly, the K-kernel had a very significantly improved fit to the data for most traits of the CIMMYT wheat, pig population, maize panel and maize connected crosses datasets. Finally, for the loblolly pine and Cornell wheat datasets, the alternative kernels did not provide a significantly improved fit over the GBLUP for most traits. Those datasets are narrow-based populations with a multifamily structure, each family being small (circular mating cross of 32 parents with an average of 13.5 individuals per family for the loblolly pine population and advanced breeding lines generated the same year for the Cornell wheat population). There are two possible explanations for this unexpected result: (1) $k$ was tested up to 15, this might not be a sufficient number of groups of individuals for those populations; however, close examination of the log-likelihood surface obtained for the grid of $k$ and the $h$ value does not support this explanation; and (2) both populations are narrow-based and thus do not present any genetic heterogeneity. Ward clustering was better than UPGMA for 38 of the 49 traits. For only three traits, (pig T3, rice FRA and PAN), the best fitting K-kernel was characterized by independent populations ($h$ = 1). Given that the results of the likelihood ratio test were supported by empirical null distributions, this demonstrates that there is significant genetic heterogeneity in the population considered and that it is trait-specific.

The log P-value of the Gaussian and K-kernel were significantly correlated across traits and datasets (correlation 0.87, P value <10^−16^), which suggests that there is some overlap in the additional information that the two kernels capture. The other pairwise correlations were not significant.

### Significant marker-trait association detection

If an alternative kernel better describes the true genetic architecture of the trait, it should better control the background effect in GWAS and increase detection power.

Type I errors with the different kernels were compared by considering the QQ plots for each trait in the rice and maize panels as is standard practice for GWAS (Additional file 2: Figures S1, S2). Under the null hypothesis, P values follow a uniform distribution and should be on the diagonal of the QQ plots with a tail indicating GWAS hits. Large deviations from the diagonal indicate large deviations from the null hypothesis and, in general, suggest a higher risk of detecting false associations [1]. In all cases, observed P values fitted a uniform distribution better (curve closer to the diagonal) when using at least one of the alternative kernels than when using the baseline model of the published GWAS study [27] (Additional file 2: Figures S1, S2). No single kernel was best to control type I errors on all traits. This is expected since Figs. 1 and 2 indicate that the kernel structure that fits the data best depended on the trait considered. Differences in the control of type I errors between models were also assessed by calculating for each trait and model, the test statistics of the Kolmogorov–Smirnov test for deviation of the P-values from a uniform distribution. Average values of the test statistics were equal to 0.161, 0.159, 0.163 and 0.161 for GBLUP, Gaussian kernel, K-Kernel and C-Kernel, respectively. These values were not significantly different based on a paired t-test. From these results, we conclude that alternative kernels control type I errors as well as GBLUP.

Statistical power was calculated as the percentage of true QTN detected at the significance threshold of 0.01 based on a sample of 5000 markers taken successively as true QTN using the same test settings as previously. On average, for the maize panel (Table 2) there was no difference in statistical power between the kernels in spite of large differences in fit to the data (Fig. 1). For the rice panel (Table 2), on average, alternative kernels provided an increase in power of about 5 % compared to the standard mixed model. Across traits, the gain in power with alternative kernels that was assessed by using a paired t test was highly significant (P values of 10^−10^, 10^−12^ and 10^−3^ for the Gaussian kernel, K-kernel and C-kernel, respectively).

**Table 2:** Average statistical power of the different kernels across QTN size and traits

Dataset	Baseline	Gaussian	K-kernel	C-kernel
Maize panel	0.700	0.699	0.700	0.700
Rice panel	0.523	0.549	0.547	0.532

All models included a Q matrix (average fraction of true QTN detected)

**Fig. 3:**
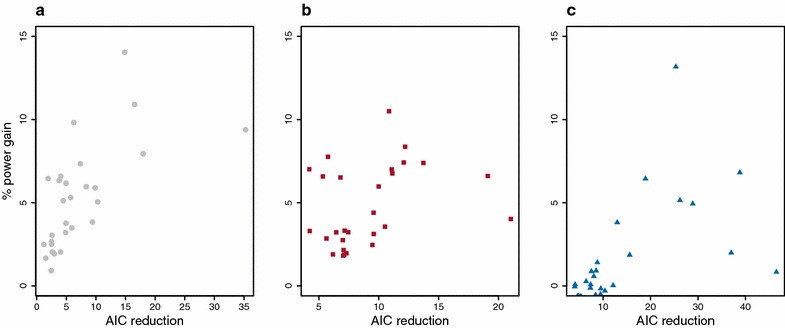
Gain in power for each trait in the rice panel with alternative kernels as a function of the reduction in AIC compared to GBLUP. **a** Gaussian kernel. **b** K-kernel. **c** C-kernel

The gain in power was similar on average for different true QTN sizes but large differences were observed between traits in the rice panel. For a few traits, gains in power were above 10 % (Fig. 3).With the Gaussian kernel and K-kernel, the power was always greater than with the base model.

With the Gaussian kernel and K-kernel (Fig. 3a, b), a significantly improved fit to the data of the alternative kernel measured by reduction of the AIC was associated with an increase in power. This trend was not as clear for the C-kernel (Fig. 3c). However, in all three cases, the correlation between power gain and AIC reduction was significant at the threshold of 0.1. This suggests that the Gaussian kernel and K-kernel can be used for routine GWAS to increase statistical power and control type I errors, and that selection of the model can be simply based on the examination of the QQ plots and the AIC to select the kernel.

### Prediction accuracy

**Fig. 4:**
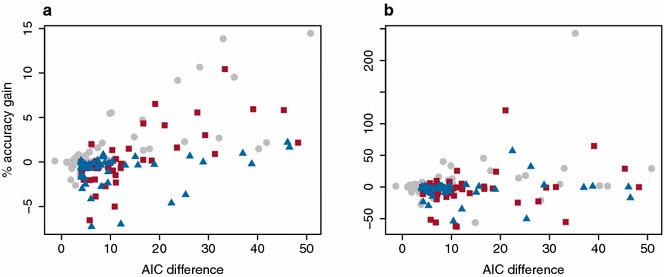
Gain in accuracy for all traits and datasets with stratified cross-validation (**a**) and leave-one-cluster-out cross-validation (**b**), for the Gaussian kernel (*gray filled circle*), the K-kernel (*red square*) and the C-kernel (*blue triangle*) for all trait-dataset combinations as a function of the reduction in AIC compared to the GBLUP

Similarly, if an alternative kernel better describes the true genetic architecture, it should also increase prediction accuracy in cross-validation. Figure 4 presents the percentage of gain in accuracies with the two cross-validation schemes as a function of the AIC reduction (complete cross-validation results are available in Additional file 1: Table S2). With stratified cross-validation, the C-kernel was never more predictive than the baseline GBLUP. For a number of traits, in stratified cross-validation, the Gaussian kernel and K-kernel performed better than GBLUP. On average, gains were small but, for some traits, gains in cross-validated accuracy were up to 10–15 %. With stratified cross-validation, as expected, a large reduction in AIC implied a gain in accuracy for both the Gaussian kernel and K-kernel (correlation higher than 0.6 and P value less than 10^−6^ in both cases) but not for the C-kernel (non-significant correlation). This provides further evidence that both the Gaussian kernel and K-kernel are not overfitting the data. With leave-one-cluster-out cross-validation, accuracies were on average much lower, especially for the most diverse datasets. The correlation between the AIC reduction and the observed gain in accuracy was less clear but still significant for the Gaussian kernel and K-kernel (correlation higher than 0.27 and P value significant at the 5 % threshold). The correlation was not significantly different from 0 for the C-kernel (P value = 0.9).

## Discussion

### Genetic heterogeneity of breeding populations

The results of the likelihood ratio tests support the hypothesis that alternative covariance estimators better describe the true genetic architecture of the trait. The tests results are supported by simulations of the null-distribution of the test statistics. The fact that this new covariance estimator better describes the true genetic architecture of the trait in some cases is evidence of significant heterogeneity of the trait architecture. The new model provides a way to investigate genetic heterogeneity and find the relevant scale to apply genomic selection or detection of marker-trait associations. The increase in GWAS power and stratified cross-validation accuracy are evidence that the alternative covariance estimators are not overfitting the data and, thus, further support the findings on genetic heterogeneity.

There are several non-exclusive explanations for the significantly improved fit to the data with alternative covariance estimators. The most obvious one is marker density, but most datasets used here have a high marker density. Another explanation is the presence of non-additive effects. Improved fit to the data with the Gaussian kernel compared to GBLUP can be explained by epistatic interactions in inbred individuals [20] but this better fit is not sufficient to conclude that epistasis is pervasive. Another explanation, which is the initial motivation for this work, is that the K-kernel accounts for uncertainty in the coefficients of ${\hat{\mathbf{G}}}$ for distantly-related individuals

### Alternative kernels for genetic evaluation and GWAS

GWAS with a mixed model is standard practice but there are few published reports of alternative kernels to control background effects. Stich et al. [30] tested a number of alternative formulae to calculate kinship for GWAS and showed that they all seemed equivalent. For two datasets, we showed that alternative kernels can provide similar or improved control of type I errors and increase power. An alternative kernel can be selected with the AIC for the Gaussian kernel and K-kernel. Because the Gaussian and K-kernel can better describe the true genetic architecture of the trait, they can better control false positive results and increase power. The K-kernel has also the advantage of helping to identify the relevant scale to apply GWAS. If for a given trait and population, $h = 1$ (several sub-populations with independent trait architecture), GWAS should probably be performed in each sub-population independently.

For the C-kernel, results suggest that the reduction in AIC is not a good predictor of an increase in power. This result contrasts with that reported in [21] for which an average gain in power of about 30 % was found for three datasets (humans, dog, maize). The main difference between their study and ours was that the number of markers was much smaller than in our datasets. In the rice dataset, the improved fit of the C-kernel to the data did not translate into increased power or prediction accuracy. Because of the high diversity of the rice dataset, the number of markers might be too small to correctly estimate the kinship, which explains the observed performance of the C-kernel. This does not exclude the possibility that the C-kernel performs well by shrinking more for low-heritability traits (Z. Zhang, personal communication). To assess the impact of marker density on C-kernel performance, we sampled 10 times a subset of markers on the maize panel dataset and computed the log-likelihood ratio test for the C-kernel. For that dataset and trait (flowering time), the test was clearly more in favor of the C-kernel at low than at full marker density (Additional file 2: Figure S3). However, in situations with a low marker density, the optimality properties of the kernel proposed in [14] are better understood.

For genomic prediction, the K-kernel or Gaussian kernel provided a small increase in accuracy in stratified cross-validation depending on the trait. Figure 4 suggests that the C-kernel was never really useful even when the reduction in AIC was large. Figure 4 also indicates that the alternative kernel should only be used when the AIC reduction is large compared to that with GBLUP. In the cross-validation reported here, model parameters were selected simply on log-likelihood. Cross-validation accuracies could be improved by using the AIC to decide which model to use.

Results of the leave-one-cluster-out cross-validation were poor, with none of the alternative kernels performing better than GBLUP. This might be expected because those kernels make additional hypotheses on relationships compared to GBLUP. If a cluster is fully removed from the training set, the model cannot reliably identify those parameters on the relationship between the training and validation sets. However, it might prove useful in across-breed predictions, if training data are available on both breeds and the focus is on prediction accuracy in a given breed. In that context, training data in the breed of interest would provide information on how informative the other breed is for the validation set.

### Implications for the design and optimization of training populations

Results from [31, 32] suggest that across-breed predictions in animal breeding are favorable when populations are closely related, marker density is high and within-breed training populations are small. If the true covariance ${\mathbf{G}}$ was available, across-breed predictions should always be beneficial. This does not seem to be the case empirically and is further confirmed by data on dairy cattle in [3], which show that the expected accuracies from the mixed model do not always match the observed accuracies. This has profound implications for the design and optimization of training populations. The decision to add distantly-related individuals to the training population, only when the training population is small and marker density is high, seems somewhat arbitrary. The K-kernel that we propose here makes that decision in a single-step analysis. If *h* = 1, it is beneficial to analyze the data in distinct sets. Otherwise, the data can be analyzed together, but the K-kernel allows the less related individuals to contribute less information to the prediction than GBLUP. The K-kernel can be used to identify an optimal training set as well as provide a way to better use distantly related individuals for training. This model is a refinement of the multi-trait model proposed by [22, 23] that deals with genetic heterogeneity between breeds and allows identification of which sets of sub-populations to use.

## Additional files

10.1186/s12711-015-0171-z Title: Complete description of the phenotypes and datasets used in this study. **Table S2.** Title: Cross-validated accuracies for all traits and datasets with the different kernels. Description: Two cross-validation schemes are used. Stratified cross-validation ensures that each validation sample is a representative sample of the complete dataset. Leave-one-cluster-out cross-validation focuses on prediction accuracies for closely related individuals with no close relatives in the training set.
10.1186/s12711-015-0171-z Title: QQ plot for the maize panel, with kinship (GBLUP) (black diamond), the Gaussian kernel (gray filled circle), the K-kernel (orange square). Description: The C-kernel result is not plotted because it was equivalent to the GBLUP for that trait (as many clusters as individuals). **Figure S2.** Title: QQ plots for the rice panel, with kinship (GBLUP) (black diamond), the Gaussian kernel (gray filled circle), the K-kernel (orange square) and the C-kernel (blue triangle). Description: The rice dataset is in Table 1 and more details on the traits are available in Additional file 1 Table S1; for all kernels, the first four principal components of the markers design matrix were fitted as fixed effect. **Figure S3.** Title: Bootstrap of the C-kernel likelihood ratio test as a function of the number of markers in the maize panel for flowering time. Description: This figure presents additional results on C-kernel sensitivity to marker density.
