# Supplementary material for: An alternative covariance estimator to investigate genetic heterogeneity in populations
Source: Genet Sel Evol. 2015 Nov 26;47:93. doi: 10.1186/s12711-015-0171-z (PMC4661961; doi:10.1186/s12711-015-0171-z)
Supplement: Supplementary file 1 — 10.1186/s12711-015-0171-z Title: Complete description of the phenotypes and datasets used in this study. Table S2. Title: Cross-validated accuracies for all traits and datasets with the different kernels. Description: Two cross-validation schemes are used. Stratified cross-validation ensures that each validation sample is a representative sample of the complete dataset. Leave-one-cluster-out cross-validation focuses on prediction accuracies for closely related individuals with no close relatives in the training set. [file 12711_2015_171_MOESM1_ESM.doc]

Table S1. Description of phenotypes and datasets used in this study

| **Dataset** | **Trait acronym** | **Trait name** | **Trait description** |
| --- | --- | --- | --- |
| Loblolly pine | HTLC | Total height to the base of the live crown | single tree plots in 8 replicates measured in cm at year 4 |
|  | BA | Branch angle average | single tree plots in 4 replicates measured in degrees at year 6 |
|  | BD | Average branch diameter | single tree plots in 4 replicates measured in cm at year 6 |
|  | BLC | basal height of the live crown | single tree plots in 8 replicates measured in cm at year 6 |
|  | CWAC | crown width across the planting beds | single tree plots in 8 replicates measured in cm at year 6 |
| Cimmyt wheat | yld1 | Grain yield | Grain yield measured in four different target environments worldwide |
|  | yld2 | Grain yield |  |
|  | yld3 | Grain yield |  |
|  | yld4 | Grain yield |  |
| Pig | T2 | Anonymous trait | Rescaled phenotype was corrected for environmental factors (e.g. year of birth or farm) of narrow sense heritability 0.16 |
|  | T3 | Anonymous trait | Rescaled weighted mean of corrected progeny phenotype of narrow sense heritability 0.38 |
| Maize panel | FT | Flowering time | Measured un growing degrees days in three locations (Ames, IA; Clayton, NC; and Aurora, NY) in 2010 |
| Maize connected crosses | GER | Giberella ear rot severity | Artificial silk channel inoculation was performed with an aggressive isolate of *F. graminearum* in two locations in Southwest Germany in 2008 and 2009 and severity scored as the ear area covered by mycelium |
|  | DON | Mycotoxin content | Deoxynivalenol (DON) content measured by near infrared spectroscopy on ground kernels |
|  | EAR | Ear length | Measured on non-infected ears in cm |
|  | KER | Kernel rows | Measured on non-infected ears |
|  | KERP | Kernels per row | Measured on non-infected ears |
| Cornell wheat | YLD | Grain yield | Grain yield in 4 locations, 2 each years in 2008 and 2009 |
|  | HT | Plant height | Plant height measure in cm in 3 locations each year in Ithaca, NY observed in 2008 and 2009 |
|  | HD | Heading date | Number of days in the year until the inflorescence is emerged in 50% of the plot in 3 locations each year in Ithaca, NY observed in 2008 and 2009 |
|  | PHS | Pre Harvest sprouting | Preharvest sprouting is the premature germination of seeds while still attached to the mother plant that decreases grain value. Measured in 3 locations each year in Ithaca, NY observed in 2008 and 2009 |
| Rice panel | FTA | Flowering time at Arkansas | Number of days until the inflorescence is 50% emerged from the flag leaf counted from the day of planting, measured at Stuttgart, Arkansas, US |
|  | FTF | Flowering time at Faridpur | Number of days until the inflorescence is 50% emerged from the flag leaf counted from the day of transplanting, measured at Faridpur, Bangladesh |
|  | FTB | Flowering time at Aberdeen | Number of days until the inflorescence is 50% emerged from the flag leaf counted from the day of planting, measured at Aberdeen, UK |
|  | FRA | Ratio of heading date (Arkansas/Aberdeen) | Days to heading in Arkansas/flowering time in Aberdeen, UK |
|  | FRF | Ratio of heading date (Faridpur/Aberdeen) | Days to heading in Faridpur/flowering time in Aberdeen, UK |
|  | FLL | Flag leaf length | Length of the flag leaf measured from leaf base to leaf tip (cm) |
|  | FLW | Flag leaf width | Width of the flag leaf measured at the widest portion of flag leaf lamina (cm) |
|  | PNM | Panicle number per plant | Average number of panicles (inflorescences) per plant |
|  | HT | Plant height | Height of plant from soil surface to tip of main panicle (inflorescence) (cm) |
|  | PNL | Panicle length | Length of panicle (inflorescence) from the base to the tip (cm) |
|  | PNB | Primary panicle branch number | Number of primary branches along the panicle (inflorescence) |
|  | SNM | Seed number per panicle | Number of seeds per panicle (inflorescence), determined by counting the number of filled spikelets along the main panicle |
|  | FPN | Florets per panicle | Average number of flowers (florets or spikelets) on main panicle (inflorescence) |
|  | PNF | Panicle fertility | Percent of spikelets that filled and produced seeds determined as the ratio of seeds per panicle/spikelets per panicle |
|  | SEL | Seed length | Length of the seed with hull (palea and lemma) |
|  | SEW | Seed width | Width of the seed with hull (palea and lemma) |
|  | SEV | Seed volume | Volume of the seed with hull (palea and lemma) |
|  | SES | Seed surface area | Surface area of the seed with hull (palea and lemma) |
|  | BSL | Brown rice seed length | Length of the unpolished rice grain (dehulled seed) |
|  | BSW | Brown rice seed width | Width of the unpolished rice grain (dehulled seed) |
|  | BSS | Brown rice surface area | Surface area of the unpolished rice grain (dehulled seed) |
|  | BSV | Brown rice volume | Volume of the unpolished rice grain (dehulled seed) |
|  | SER | Seed length/width ratio | Ratio of seed length/ seed width (with hull) |
|  | BSR | Brown rice length/width ratio | Ratio of unpolished rice grain length/grain width (dehulled seed) |
|  | BLA | Blast resistance | Disease severity on rice leaf caused by the fungus *Pyricularia oryzae*. For blast resistance evaluation, the accessions were inoculated with a mixture of the U.S. blast races, in a blast screening nursery at Beaumont, TX during 2009 and 2010. The disease severity was scored on a “0” (no disease) to “9” (dead) scale when the plants were three to four weeks old. |
|  | AMY | Amylose content | Amount of amylose present in the milled grains. Estimation of amylose content of each accession was done in the Rice Quality Lab, USDA-ARS, Stuttgart, Arkansas |
|  | ALK | Alkali spreading value | Observed by placing six milled-rice kernels in 10ml 1.7% KOH in a shallow container for 23hrs at 30 degree (°C) temperature and scoring for the extent of digestion of the starch based on its level of intactness. Measure for alkali digestion is inversely proportional to the gelatinization temperature, e.g. if alkali digestion is low, the gelatinization temperature is high. Estimation of ASV of each accession was done in the Rice Quality Lab, USDA-ARS, Stuttgart, Arkansas |
|  | PRO | Protein content | Protein content in brown rice (dehulled grain with pericarp). Estimation of protein content of each accession was done in the Rice Quality Lab, USDA-ARS, Stuttgart, Arkansas |

Table S2 Cross validated accuracies for all traits and datasets with the different kernels. Two cross-validation schemes are used. Stratified cross-validation ensures that each validation sample is a representative sample of the complete dataset. Leave one cluster out cross-validation focus on prediction accuracies within closely related individuals with no close relatives in the training set.

|  |  | **Stratified cross-validation** | | | | **Leave one cluster out cross-validation** | | | |
| --- | --- | --- | --- | --- | --- | --- | --- | --- | --- |
| **dataset** | **trait** | **G-kernel** | **Gaussian** | **K-Kernel** | **C-Kernel** | **G-kernel** | **Gaussian** | **K-Kernel** | **C-Kernel** |
| Loblolly pine | HTLC | 0.46 | 0.46 | 0.45 | 0.46 | 0.46 | 0.46 | 0.45 | 0.45 |
|  | BA | 0.51 | 0.51 | 0.51 | 0.51 | 0.5 | 0.5 | 0.5 | 0.51 |
|  | BD | 0.28 | 0.27 | 0.27 | 0.27 | 0.25 | 0.24 | 0.27 | 0.25 |
|  | BLC | 0.49 | 0.49 | 0.48 | 0.48 | 0.42 | 0.42 | 0.42 | 0.41 |
|  | CWAC | 0.47 | 0.47 | 0.47 | 0.47 | 0.43 | 0.43 | 0.44 | 0.42 |
| Cimmyt wheat | yld1 | 0.5 | 0.57 | 0.53 | 0.5 | 0.13 | 0.17 | 0.21 | 0.12 |
|  | yld2 | 0.48 | 0.49 | 0.47 | 0.47 | 0.3 | 0.28 | 0.26 | 0.28 |
|  | yld3 | 0.39 | 0.43 | 0.41 | 0.37 | 0.12 | 0.1 | 0.09 | 0.19 |
|  | yld4 | 0.46 | 0.53 | 0.51 | 0.47 | 0.09 | 0.12 | 0.04 | 0.08 |
| Pig | T2 | 0.49 | 0.5 | 0.5 | 0.5 | 0.2 | 0.19 | 0.15 | 0.2 |
|  | T3 | 0.32 | 0.33 | 0.34 | 0.32 | 0.09 | 0.1 | 0.11 | 0.08 |
| Maize panel | FT | 0.9 | 0.9 | 0.9 | 0.9 | 0.62 | 0.63 | 0.63 | 0.62 |
| Maize connected crosses | GER | 0.76 | 0.76 | 0.76 | 0.75 | 0.58 | 0.57 | 0.57 | 0.57 |
|  | DON | 0.79 | 0.81 | 0.81 | 0.79 | 0.56 | 0.57 | 0.56 | 0.55 |
|  | EAR | 0.58 | 0.59 | 0.6 | 0.58 | 0.48 | 0.51 | 0.48 | 0.48 |
|  | KER | 0.82 | 0.83 | 0.82 | 0.82 | 0.64 | 0.66 | 0.64 | 0.64 |
|  | KERP | 0.41 | 0.45 | 0.43 | 0.41 | 0.32 | 0.36 | 0.31 | 0.32 |
| Cornell wheat | YLD | 0.42 | 0.42 | 0.42 | 0.42 | 0.32 | 0.32 | 0.32 | 0.32 |
|  | HT | 0.55 | 0.58 | 0.55 | 0.52 | 0.29 | 0.33 | 0.28 | 0.27 |
|  | HD | 0.47 | 0.47 | 0.44 | 0.47 | 0.33 | 0.33 | 0.3 | 0.33 |
|  | PHS | 0.55 | 0.55 | 0.56 | 0.53 | 0.4 | 0.41 | 0.41 | 0.31 |
| Rice panel | FTA | 0.68 | 0.68 | 0.68 | 0.68 | 0.3 | 0.29 | 0.28 | 0.3 |
|  | FTF | 0.52 | 0.51 | 0.51 | 0.51 | 0.12 | 0.11 | 0.11 | 0.12 |
|  | FTB | 0.59 | 0.59 | 0.58 | 0.59 | 0.22 | 0.17 | 0.09 | 0.2 |
|  | FRA | 0.53 | 0.54 | 0.52 | 0.53 | 0.18 | 0.15 | 0.09 | 0.18 |
|  | FRF | 0.48 | 0.49 | 0.45 | 0.47 | 0.1 | 0.05 | 0.04 | 0.07 |
|  | FLL | 0.53 | 0.52 | 0.53 | 0.53 | 0.18 | 0.13 | 0.16 | 0.17 |
|  | FLW | 0.76 | 0.76 | 0.76 | 0.76 | 0.35 | 0.36 | 0.35 | 0.33 |
|  | PNM | 0.82 | 0.82 | 0.82 | 0.82 | 0.21 | 0.22 | 0.22 | 0.28 |
|  | HT | 0.77 | 0.77 | 0.76 | 0.77 | 0.17 | 0.17 | 0.19 | 0.17 |
|  | PNL | 0.66 | 0.67 | 0.66 | 0.66 | 0.02 | 0.03 | 0.03 | 0.01 |
|  | PNB | 0.64 | 0.65 | 0.64 | 0.62 | 0.24 | 0.28 | 0.22 | 0.25 |
|  | SNM | 0.57 | 0.57 | 0.57 | 0.53 | 0.22 | 0.21 | 0.21 | 0.18 |
|  | FPN | 0.66 | 0.67 | 0.66 | 0.67 | 0.29 | 0.29 | 0.31 | 0.28 |
|  | PNF | 0.54 | 0.57 | 0.57 | 0.52 | 0.12 | 0.16 | 0.15 | 0.06 |
|  | SEL | 0.76 | 0.76 | 0.76 | 0.76 | 0.43 | 0.43 | 0.44 | 0.44 |
|  | SEW | 0.84 | 0.84 | 0.84 | 0.84 | 0.4 | 0.41 | 0.38 | 0.41 |
|  | SEV | 0.82 | 0.83 | 0.82 | 0.82 | 0.38 | 0.4 | 0.31 | 0.37 |
|  | SES | 0.79 | 0.79 | 0.79 | 0.79 | 0.38 | 0.38 | 0.34 | 0.38 |
|  | BSL | 0.81 | 0.8 | 0.8 | 0.8 | 0.51 | 0.51 | 0.52 | 0.49 |
|  | BSW | 0.85 | 0.85 | 0.85 | 0.85 | 0.42 | 0.46 | 0.4 | 0.44 |
|  | BSS | 0.78 | 0.78 | 0.77 | 0.78 | 0.39 | 0.41 | 0.34 | 0.38 |
|  | BSV | 0.83 | 0.84 | 0.83 | 0.83 | 0.43 | 0.47 | 0.38 | 0.42 |
|  | SER | 0.8 | 0.8 | 0.8 | 0.8 | 0.39 | 0.41 | 0.38 | 0.4 |
|  | BSR | 0.83 | 0.83 | 0.83 | 0.83 | 0.47 | 0.48 | 0.46 | 0.47 |
|  | BLA | 0.69 | 0.7 | 0.69 | 0.68 | 0.36 | 0.36 | 0.34 | 0.35 |
