# Supplementary material for: An alternative covariance estimator to investigate genetic heterogeneity in populations
Source: Genet Sel Evol. 2015 Nov 26;47:93. doi: 10.1186/s12711-015-0171-z (PMC4661961; doi:10.1186/s12711-015-0171-z)
Supplement: Supplementary file 2 — 10.1186/s12711-015-0171-z Title: QQ plot for the maize panel, with kinship (GBLUP) (black diamond), the Gaussian kernel (gray filled circle), the K-kernel (orange square). Description: The C-kernel result is not plotted because it was equivalent to the GBLUP for that trait (as many clusters as individuals). Figure S2. Title: QQ plots for the rice panel, with kinship (GBLUP) (black diamond), the Gaussian kernel (gray filled circle), the K-kernel (orange square) and the C-kernel (blue triangle). Description: The rice dataset is in Table 1 and more details on the traits are available in Additional file 1 Table S1; for all kernels, the first four principal components of the markers design matrix were fitted as fixed effect. Figure S3. Title: Bootstrap of the C-kernel likelihood ratio test as a function of the number of markers in the maize panel for flowering time. Description: This figure presents additional results on C-kernel sensitivity to marker density. [file 12711_2015_171_MOESM2_ESM.doc]

Figure S1 QQ plot for the maize panel, with kinship (G-kernel) (black diamond), the Gaussian kernel (gray filled circle), the K-kernel (orange square).


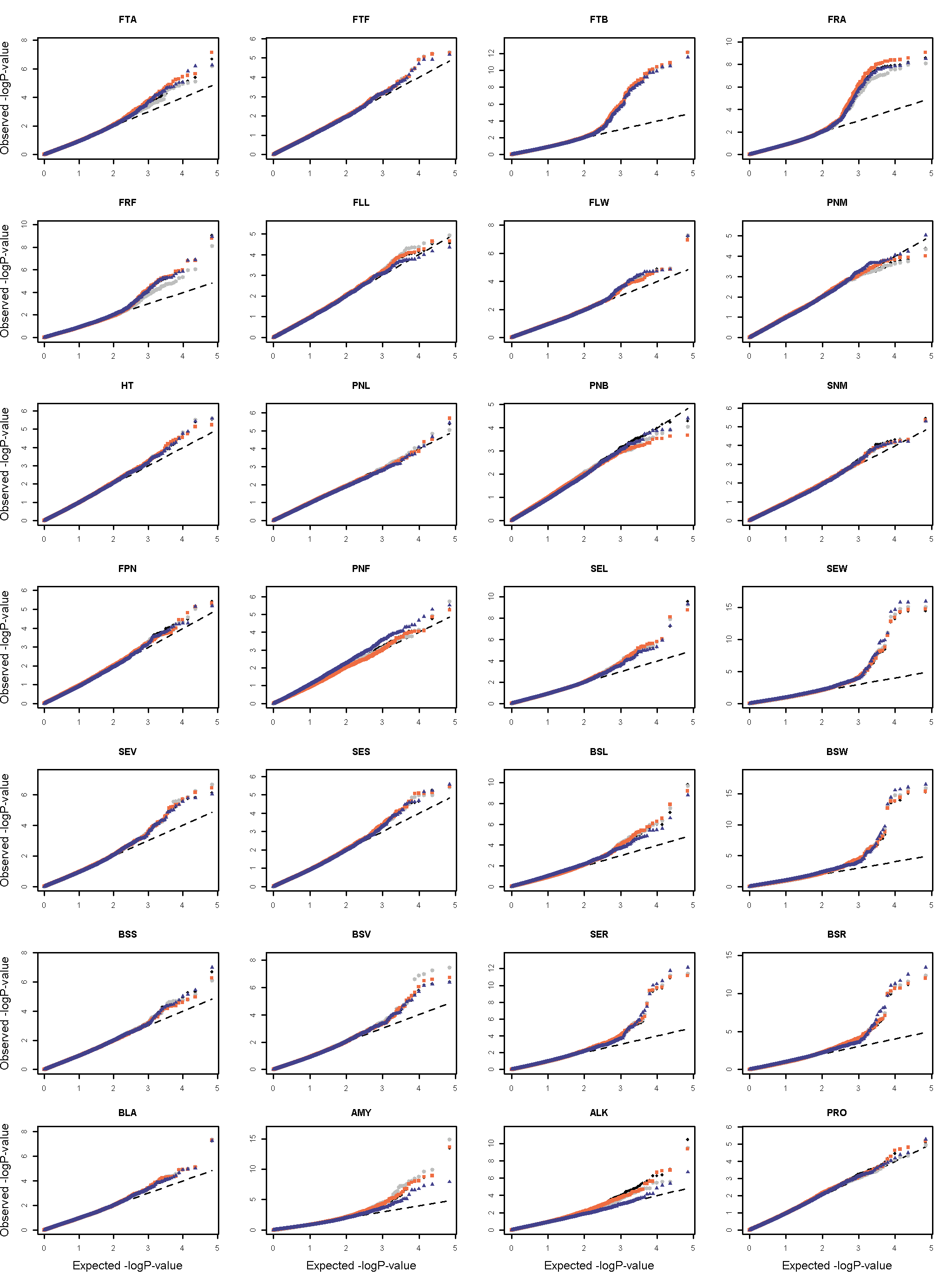


Figure S2 QQ plots for the rice panel, with kinship (G-kernel) (black diamond), the Gaussian kernel (gray filled circle), the K-kernel (orange square) and the C-kernel (blue triangle).

Figure S3 Bootstrap of the C-kernel Likelihood ratio test as a function of the number of markers, maize panel flowering time
